# Fixed pitch size small-sided games in young soccer players: effects of different age categories and competitive levels on the physical and physiological responses

**DOI:** 10.5114/biolsport.2025.144298

**Published:** 2024-10-23

**Authors:** Bilel Cherni, Hamza Marzouki, Okba Selmi, Bruno Gonçalves, Karim Chamari, Yung-Sheng Chen, Anissa Bouassida

**Affiliations:** 1 Higher Institute of Sport and Physical Education of Ksar Saïd, University of Manouba, Manouba, Tunisia; 2 Research Unit: Sport Sciences, Health and Movement, University of Jendouba, El Kef, Tunisia; 3 High Institute of Sport and Physical Education of Kef, University of Jendouba, El Kef, Tunisia; 4 Departamento de Desporto e Saúde, Escola de Saúde e Desenvolvimento Humano, Universidade de Évora, Évora, Portugal; 5 Comprehensive Health Research Centre (CHRC), Universidade de Évora, Évora, Portugal; 6 Portugal Football School, Portuguese Football Federation, Oeiras, Portugal; 7 Naufar, Wellness and Recovery Center, Doha, Qatar; 8 Department of Exercise and Health Sciences, University of Taipei, Taipei, Taiwan; 9 Exercise and Health Promotion Association, New Taipei City, Taiwan; 10 High Performance Unit, Chinese Taipei Football Association, New Taipei City, Taiwan

**Keywords:** Youth, Football training, GPS devices, Time-motion variables, Chronological age

## Abstract

We compared the physical and physiological responses of young soccer players of different ages U13 (Under 13 years), U15, and U17 and competitive levels (elite and sub-elite) during fixed-pitch size smallsided games (SSGs) performed under different formats. In a cross-sectional design, seventy-two male players (12 players in each group) performed 3-a-side and 4-a-side SSGs with a fixed-pitch size (40 × 20 m). The total distance covered (TD), peak velocity (Vpeak), the distances covered at different running speed zones (0 to < 7.0, 7.0 to < 14.0, 14.0 to < 18.0, and ≥ 18.0 km.h^−1^), peak heart rate (HRpeak), HRmean, expressed as percentage of the theoretical HR_max_Theo, and blood lactate concentration post-SSGs (BLa) were recorded. Players in the 3-a-side SSGs covered more TD, distances covered at different speeds and Vpeak than those of the 4-a-side SSGs across all age categories of both competitive levels (p < 0.05). The 3-a-side SSGs induced higher BLa in all elite groups (p < 0.05), and U13-sub-elite players (p=0.004). HR_peak_(%HR_max_Theo) and HR_mean_(%HR_max_Theo) were greater in the 4-a-side SSGs than the 3-a-side SSGs in most age categories of both competitive levels (p < 0.05). In the zone-3, the U13- and U15-elite covered more distance than U17 in both formats. In both formats, elite players covered larger TD and distances at zone-1 and zone-4 sub-elite players (p < 0.05). BLa was higher in U17-sub-elite compared to their elite counterparts in the 4-a-side SSGs. Our study shows that 3-a-side SSGs are physically more demanding than 4-a-side, especially for elite players. Tailoring training to age and competitive level is crucial for optimising player development.

## INTRODUCTION

Soccer performance involves fitness, technical-, and tactical skills [1, 2, 3]. These factors collectively influence outcomes during training and matches [4]. Given soccer’s intermittent nature with high-intensity actions, training must develop the specific bioenergetic demands of intermittent efforts [5, 6]. High-intensity interval training (HIIT), particularly through small-sided games (SSGs), addresses key game dimensions while enhancing soccer-specific fitness [7]. Indeed, SSGs effectively develop fitness and skills [8] by simulating soccer playing conditions, improving aerobic fitness, technical skills, and tactical awareness at the same time [9]. Evidence shows SSGs are as effective as running-based exercises in fitness development [10].

Previous studies have explored task constraints to understand the influence of altering SSG characteristics [11], such as the number of players per team [12] and pitch size variations [13]. The 3-a-side SSG format often increases physical demands when it involves a higher relative area per player, promoting more frequent high-intensity actions [8]. However, designing a 3-a-side game with a lower relative area is also possible, which may alter these demands [8]. Conversely, larger formats like the 4-a-side can foster greater tactical engagement and aerobic conditioning when the relative area per player is increased, resulting in more extensive player distribution on the pitch. However, the specific pitch size can be adjusted to alter these physical responses, allowing coaches to tailor the demands based on training objectives [9]. Several previous studies have investigated the different SSG formats with pitch size variation in order to respect the ‘’space-by-player’’ variable [14, 15]. While it is often assumed that smaller team sizes such as the 3-a-side format are more physically demanding, this assumption does not hold when the relative area per player is balanced. Larger relative areas result in higher physical and physiological demands, regardless of the number of players involved [15]. To the best of our knowledge, only a few studies have examined the influence of team size on physiological responses and activity demands of SSGs with fixed pitch size in young recreational adult players [16]. Therefore, we believe that scientists should investigate all the options (e.g. changing or fixed pitch sizes) to offer more evidence-based training tools to the coaches. Specifically, Stojanovic et al. [16] have reported no significant differences in HR, BLa, and physical performances between 3- and 4-a-side SSGs played on fixed pitch size. This finding contradicts the general expectation based on previous literature that smaller areas per player, such as in 3-a-side formats, would be more physically demanding [15]. The lack of studies examining the influence of team size on the physiological responses and activity demands of SSGs with fixed pitch size in youth populations is a significant gap. This limits our understanding of how different SSG formats affect young players, who have different physiological and developmental needs compared to adults. Without this knowledge, coaches and trainers may not optimize training protocols for youth players, potentially affecting their development and performance. In this regard, understanding the nuances and potential exceptions is essential, ensuring that training methods are evidence-based and tailored to actual findings. This examination will offer coaches precise tools and strategies for training, ultimately benefiting player development and performance. Quantifying internal physiological responses and external activity demands provides insight into the physical workloads imposed on players during SSGs [16]. The external demands relative to team size in youth soccer SSGs in the literature [17, 18] yielded inconclusive outcomes, indicating a need for further investigations with a broader range of metrics. To date, the physiological responses and activity demands during 4-a-side SSGs have not been compared to other formats in youth soccer players of different competitive levels. Thus, analyzing the demands of 4-a-side SSGs against other game formats is crucial for better training practical applications.

In youth soccer training, players are often grouped based on skill level and competitive experience [19]. High-level athletes encounter more demanding training and competition environments, promoting their development through intense gameplay [20]. Conversely, players at lower competitive levels face challenges like limited competition opportunities [21]. For instance, Studies show that higherlevel athletes often exhibit superior physical capacities, such as speed, endurance, and strength, compared to lower-level counterparts [22, 23]. Similarly, the competitive level also impacts technical and tactical skills, with more competitive environments fostering better game understanding and refined technical abilities [24]. The current study aims to fill this gap by analyzing the physiological and physical activity demands of different SSG formats in youth soccer players of different competitive levels. Although technical and tactical variables are crucial, they are not the focus of this investigation, which instead centres on quantifying the physical and physiological responses to provide insights into training demands.

In terms of physical performance in youth, older players (Under; U15) cover greater distances during matches compared to younger players (U13) [25]. U13 players also show a more pronounced decline in physical responses from the first to the second half of a match [25], likely due to lower fitness [11]. Additionally, older players exhibit superior spatial occupation in SSGs [13]. For instance, Santos et al. [13] found significant variations in external load experienced by youth players in SSGs based on age, indicating different physical demands for younger (U13) versus older players (U15, U17). Similarly, López-Fernández et al. [12] also reported distinct responses among U14, U16, and U18 players in different SSG formats. This finding suggests that SSGs impose different training loads across age groups, highlighting the need to avoid general conclusions about their demands without considering age-specific variations [11].

The aim of our study was to investigate the effects of the competitive level and age group on the physical performance and physiological responses of youth soccer players to same-size pitch SSGs. We hypothesized to find (i) higher physical and physiological demands in the 3-a-side SSG compared to the 4-a-side format, (ii) that elite players will demonstrate superior physical and physiological responses compared to sub-elite players across all age categories, and (iii) that older age categories’ players will perform better in terms of physical performance metrics compared to the younger players.

## MATERIALS AND METHODS

### Participants

Prior to the recruitment procedure, a sample size estimation was conducted using statistical software (G*Power software, version 3.1.9.4, University of Kiel, Kiel, Germany) [26]. Given the study design (analysis of variance (ANOVA) test with repeated measures, within-between interaction), the effect sizes considered to generate the sample size estimation were attained based on tabled data from previous research [27]. The results established a need for 10 participants per group (f = 0.25 and actual power = 82.46%) to detect differences with an assumed Type-I error of 0.05 and a Type-II error rate of 0.20 (statistical power = 80%). Thus, seventy-two Tunisian male youth soccer players (Elite: competing in the top tier of the first youth league, n = 36; sub-elite: participating in the fourth youth league, n = 36) enrolled in the Tunisian Football Federation volunteered to participate in this study. The usual micro-cycle training for elite and sub-elite cohorts consists of five (~70 to 100 min each session) and three sessions a week (~60 to 80 min each session), respectively, with a weekly match scheduled on Sunday. Participants were categorised into 3 subgroups according to age (12 elite and 12 sub-elite for each of the U13, U15 and U17 age categories). To be eligible to participate in the study, players had to meet the following inclusion criteria: (a) being free from (i) severe musculoskeletal injuries for at least one year and (ii) mild to moderate injury for the month, preceding the study [28], and (b) having a minimum of 2 years of soccer experience and regularly engage in the club’s training routines. Participant characteristics per competitive level and age are illustrated in Table 1. This study received institutional ethics approval (approval number: 004/2020; date of approval: February 11, 2020) and was conducted by the Declaration of Helsinki. Volunteers provided written, informed consent forms before starting their participation in the trial.

**TABLE 1:** Participants’ physical characteristics (mean ± SD) (n =72).

		Age (years)	Height (cm)	Body weight (kg)	BMI (kg · m^-^2^^)
**Elite**	U13 (n=12)	12.1 ± 0.6	148.2 ± 7.5	39.7 ± 6.4	18.6 ± 1.3
	U15 (n=12)	14.4 ± 0.9	164.8 ± 7.6	51.8 ± 6.7	19.07 ± 2.4
	U17 (n=12)	16.1 ± 1.1	176.6 ± 6.7	86.2 ± 3.2	21.46 ± 2.6

**Sub-Elite**	U13 (n=12)	12.2 ± 0.6	149.2 ± 5.9	39.3 ± 5.7	17.44 ± 1.3
	U15 (n=12)	14.0 ± 0.5	163.8 ± 9.4	50.3 ± 4.5	18.6 ± 1.3
	U17 (n=12)	16.1 ± 0.6	175.8 ± 7.0	65.6 ± 4.9	21.31 ± 1.8

Note: SD: standard deviation; U13: under 13; U15: under 15: U17: under 17; BMI: body mass index

### Experimental Approach to the Problem

**FIG. 1:**
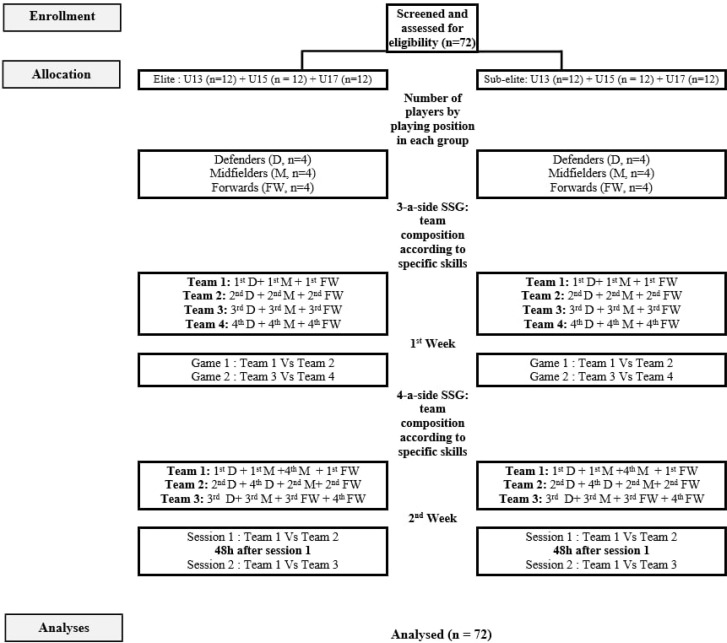
Team composition procedures within each category.

The current study adopted a cross-sectional design to explore the impact of age category and competitive level on male youth soccer players’ physical and physiological responses during SSGs performed on different formats. The study was conducted during the soccer in-season period (February 2022, while the seasons started in September 2021) and lasted 3 weeks. The first week was devoted to (i) assessing the anthropometric parameters [body mass was measured to the nearest 0.1 kg using a digital scale (OHAUS, Florhman Park, NJ, USA), body height was measured to the nearest 0.01 m, and body mass index (BMI) was calculated (kg · m-^2^)]; and (ii) to familiarise participants with wearing the global positioning system (GPS) devices and HR monitors (Polar Team Pro, Kempele, Finland), as well as testing training on each of the SSG formats (i.e., 3- and 4-a-side). The remaining two weeks were used to administer each SSG format [one session in the 1^st^ week (Tuesday) and 2 sessions in the 2^nd^ week (Tuesday and Thursday)]. Based on specific soccer skills, each player was ranked by the coach concerning his abilities in passing, close ball control, shooting, and game sense using a 5-point Likert scale (1 = “outstanding,” to 5 = “below average”) [29]. Within each age and competitive level group, teams were arranged according to the ranking in the specific soccer skill results [29] and game position (i.e., defenders, midfielders, forwards) (Figure 1). For example, in the first 3-a-side confrontation, team 1 consisted of the best forward, best midfielder, and best defender, while team 2 consisted of the second-best forward, second-best midfielder, and second-best defender. This counterbalanced procedure allowed close technical performance conditions between teams [30]. A draw designated all the confrontations between the teams. Additionally, the order of the SSG formats (3-a-side and 4-a-side) was randomized to reduce order bias. The procedures regarding the teams’ composition are described in Figure 1. All SSGs sessions were conducted with a minimum interval of 48 hours after the latest weekly match or before the next game or intensive training session to minimise the impact of fatigue on the study outcomes. The study assessments were carried out under standardized conditions to ensure consistency across measurements.

### Procedure

#### Small-Sided Games

All SSG sessions were conducted under similar environmental conditions (Temperature: 14–18°C, humidity: ~ 74%). The games were played at the same time of the day, at the beginning of the training session (16 to 18 h), with each session being played on the same playing area (i.e., a regular turf soccer pitch). Participants performed structured 3- and 4-a-side + two goalkeepers formats [16]. Both SSG formats comprised two periods (halves) lasting 4 min each, interspersed with one minute of passive recovery. The dimensions of the pitch area were constant in the two-game formats 20 m (width) × 40 m (length) to avoid the effect of pitch size [31]; thus, the area per player was ~133 and 100 m^2^ for the 3-a-side and 4-a-side formats, respectively. The size of the goals was 3 m wide and 1.5 m high [31], and players were instructed to strive for victory by scoring more goals than the opposing team. Each SSG session started with a dynamic and engaging 20-min warm-up routine, including cardiovascular exercises, agility drills, and dynamic stretches to enhance flexibility and coordination. The warm-up exercises concluded with 3 sets of 10-m acceleration sprints, allowing participants to transition from dynamic movements to explosive bursts of sprint. Time-motion physical performance and HR, and BLa responses were tracked. Two coaches circled the pitch, promptly supplying new balls for uninterrupted play. Players received standardized instructions to strive for victory by scoring more goals than the opposing team, without any additional tactical or technical guidance from the coaches during the games.

### Time-motion Physical Responses

GPS units (SPI ProX; GPSports, Canberra, Australia) were used to record data on the physical demands of each SSG bout, capturing data directly at 10 Hz. The efficacy of 10-Hz GPS technology has been previously established as dependable and accurate in evaluating movement profiles in team sports [32]. The variables analysed included the total distance covered (TD), peak velocity (Vpeak) (m · s^−1^), and the distances covered at specific speed zones [18] (i.e., stationary/walking (zone-1): 0 to < 7.0 km · h^−1^, low-intensity running (zone-2): 7.0 to < 14.0 km · h^−1^, medium-intensity running (zone-3): 14.0 to < 18.0 km · h^−1^, and high-intensity running (zone-4): ≥ 18.0 km · h^−1^; all measured in meter. Data extraction was performed using the Team AMS R1 2016 software, with players consistently wearing the same GPS device across all data collection sessions, ensuring data uniformity and accuracy.

### Heart Rate Response

Heart rate was recorded at 1-Hz using HR monitors (Polar®, FS1, Kempele, Finland). HR data of recovery periods were excluded from the analysis. The variables in this investigation were HRpeak, the highest recorded value during the two bouts of SSGs, and HRmean, the average of HR collected from the two halves. To standardize the intensity of exercise across different age groups, HR values were expressed as a percentage of the theoretical maximum heart rate (HR_max_). The theoretical maximum heart rate was calculated using the following formula: HR_max_ = 208 – (0.7 × age) [33]. This method ensures that the reported heart rate data accurately reflect the relative exercise intensity for each player, accounting for individual differences in age.

### Blood Lactate Concentration

Blood lactate concentrations were assessed at 3 minutes post-SSGs using a Lactate Pro analyzer (Arkray Inc., Kyoto, Japan); this method allowed for precise determination of lactate levels, offering valuable insights into the metabolic responses during the recovery phase [34].

### Statistical Analyses

Descriptive data were presented as means ± standard deviation (SD). Before inferential statistics, the Shapiro–Wilk and Levene’s tests were performed to analyse whether the variables followed a normal distribution and verify the homogeneity of the variances, respectively. A 3-way analysis of variance with repeated measures-ANOVA [age (U13, U15 and U17) × competitive level (sub-elite and elite) × game format (3- and 4-a-side) were applied to test age and competitive level effects on the dependent variables in response to SSGs game formats. When significant main effects or interactions were achieved, Bonferroni post hoc analyses were performed to locate the pairwise. To estimate the strength of significant findings, effect sizes (ES) were determined by converting the partial eta squared to Cohen’s d [35]. Effect size values were interpreted as follows: < 0.20 represents a trivial effect; 0.20 to < 0.50: small effect; 0.50 to < 0.80: intermediate effect; and ≥ 0.80: large effect [35]. The statistical analyses were conducted using SPSS software v.26 for Windows (IBM Corp, Armonk, N.Y., USA), and the significance level was established at p < 0.05.

## RESULTS

The normality of the data and the homogeneity of variance were confirmed.

For total distance covered during the SSGs, there was a statistical interaction between format, age and level (Tables 2), with U13 performing less than U15 and U17 in the 3-a-side (p = 0.023 and p = 0.009; ES = 0.877, and ES = 1.027, respectively), and less than U17 in the 4-a-side (p = 0.035; ES = 0.897) for sub-elite players. When players were pooled across age, the performances were significantly higher in elite than sub-elite in either U13 (3-aside: p < 0.0001 and ES = 1.356; 4-a-side: p < 0.0001 and ES = 0.706) or U15 (3-a-side: p < 0.0001 and ES = 1.907; 4-aside: p < 0.0001, and ES = 1.861). Within age, the 3-a-side performances were higher than the 4-a-side performances in either elite or sub-elite groups (p < 0.05) (Table 2).

**TABLE 2:** Total distance covered and maximum velocity responses during different soccer small-sided game formats in elite and subelite players (n=72).

Variables	Age	Level	Format		Within sub-group comparison p (ES)	ANOVA

			3-a-side	4-a-side		
TD (m)	U13	Elite	925.8 ± 37.2†	913.0 ± 41.1	< 0.0001 (0.326)	Format: F_1.66_ = 216.795; p < 0.0001; ES = 1.781 Age: F = 6.336; p = 0.003; ES = 0.393 Level: F_1.66_ = 33.775; p < 0.0001; ES = 0.694 Format × Age: F_2.66_ = 0.821; p = 0.444; ES=0 Format × Level: F_1.66_ = 0.927; p = 0.339; ES = 0 Age × Level: F_2.66_ = 1.667; p = 0.197; ES = 0.139 Format × Age × Level: F_2.66_ = 5.774; p = 0.005; ES = 0.372
		Sub-elite	852.6 ± 66.8†	847.3 ± 65.9	< 0.0001 (0.079)	

	U15	Elite	964.3 ± 39.5†	952.9 ± 40.3	< 0.0001 (0.284)	
		Sub-elite	897.8 ± 29.5†	886.8 ± 30.7	0.003 (0.363)	

	U17	Elite	932.2 ± 33.1†	923.7 ± 33.7	< 0.0001 (0.255)	
		Sub-elite	903.2 ± 20.2†	890.8 ± 19.1	< 0.0001 (0.632)	

V_peak_ (m · s_-1_)	U13	Elite	16.8 ± 0.9†	16.3 ± 1.1	0.048 (0.56)	Format: F_1.66_ = 73.365; p < 0.0001; d = 1.031 Age: F_2.66_ = 3.073; p = 0.053; d = 0.245 Level: F_1.66_ = 3.641; p = 0.061; d = 0.197 Format × Age: F_2.66_ = 1.831; p = 0.168; d=0.155 Format × Level: F_1.66_ = 0.675; p = 0.414; d=0 Age × Level: F_2.66_ = 0.027; p = 0.974; d = 0. Format × Age × Level: F_2.66_ = 0.014; p = 0.986; d = 0
		Sub-elite	16.6 ± 1.2†	15.8 ± 1.2	0.005 (0.70)	

	U15	Elite	17.7 ± 1.1†	16.5 ± 1.2	< 0.0001 (1.002)	
		Sub-elite	17.3 ± 0.9†	16.0 ± 1.2	0.003 (1.264)	

	U17	Elite	17.8 ± 1.0†	16.8 ± 1.2	< 0.0001 (0.916)	
		Sub-elite	17.3 ± 0.9†	16.2 ± 0.9	< 0.0001 (1.285)	

Note: Values are given as means ± SD; TD: total distance covered; V_peak_: peak velocity; for other abbreviations see Table-1; ES: effect size;

† : Significantly different from 4-a-side.

There was a format effect for Vpeak, with 3-a-side performances being higher than 4-a-side performances in either elite or subelite groups (all p < 0.05) (Table 2).

For zone-3, there was a statistical interaction between format, age and level (Tables 3), with U13 and U15 resulting in higher performances than U17 in either 3-a-side (p = 0.001 and p < 0.0001; ES = 1.771 and ES = 1.889, respectively) or 4-a-side (p = 0.01 and p < 0.0001; ES = 1.712 and ES = 1.928, respectively). Within age, the 3-a-side performances were higher than the 4-a-side performances in either elite (across all age categories: p < 0.05) or subelite groups (U15 and U17: p < 0.05) (Table 3). A significant interaction between format × level was found for zone-1, with 3-a-side showing higher values than 4-a-side in either U13 (p < 0.05 for elite), U15 and U17 (all p < 0.05 for both competitive levels) (Table 3). When players were pooled across age, zone-1 performances were significantly higher in elite level than sub-elite level in either U13 (3-a-side: p < 0.0001 and ES = 1.966; 4-a-side: p < 0.0001 and ES = 1.623), U15 (3-a-side: p < 0.0001 and ES = 2.294; 4-aside: p < 0.0001 and ES = 2.107), or U17 (3-a-side: p < 0.0001 and ES = 2.425; 4-a-side: p < 0.0001 and ES = 2.438) (Table 3). There was a format effect for zones 2 and 4 (Table 3), with 3-a-side performances being higher than 4-a-side performances in either elite (zone-2 across all age categories: p < 0.05; zone-4 in U15: p = 0.012) or sub-elite (zone-2 across all age categories: p < 0.05) (Table 3). When players were pooled across age, the performances were significantly higher in elite than sub-elite (p = 0.039 and ES = 0.393) in zone-4 (Table 3). A main effect for age was observed in zone-2 with U15 and U17 covering more distance than U13 (p = 0.017 and p = 0.032; ES = 0.82 and ES = 0.785, respectively).

**TABLE 3:** Distances covered at different speed zones during different soccer small-sided game formats in elite and sub-elite players (n = 72).

Variables	Age	Level	Format		Within sub-group comparison p (ES)	ANOVA

			3-a-side	4-a-side		
Zone 1 (m)	U13	Elite	334.7 ± 34.3†	328.0 ± 38.3	< 0.0001 (0.176)	Format: F_1.66_ = 62.055; p < 0.0001; ES = 0.947 Age: F_2.66_ = 1.020; p = 0.366; ES = 0.24 Level: F_1.66_ = 78.360; p < 0.0001; ES = 1.067 Format × Age: F_2.66_ = 0.556; p = 0.576; ES = 0 Format × Level: F_1.66_ = 4.153; p = 0.046; ES = 0.215 Age × Level: F_2.66_ = 0.034; p = 0.967; ES = 0 Format × Age × Level: F_2.66_ = 2.843; p = 0.065; ES = 0.231
		Sub-elite	283.7 ± 13.0	282.2 ± 12.0	NS		

	U15	342.8 ± 29.7†	338.5 ± 31.9	< 0.0001 (0.138)		
		Sub-elite	292.1 ± 9.7†	288.5 ± 10.6	0.002 (0.353)	

	U17	Elite	340.4 ± 23.3†	337.5 ± 23.1	0.011 (0.126)	

		Sub-elite	293.6 ± 14.3†	290.7 ± 14.4	0.011 (0.204)	

Zone 2 (m)	U13	Elite	404.3 ± 14.7†	400.8 ± 13.1	< 0.0001 (0.25)	Format: F_1.66_ = 102.991; p < 0.0001; ES = 1.225 Age: F_2.66_ = 5.067; p = 0.009; ES = 0.334 Level: F_1.66_ = 2.177; p = 0.145; ES = 0.131 Format × Age: F_2.66_ = 0.273; p = 0.762; ES = 0 Format × Level: F_1.66_ = 0.238; p = 0.628; ES = 0 Age × Level: F = 0.238; p = 0.821; ES = 0 Format × Age × Level: F_2.66_ = 1.157; p = 0.321; ES = 0.067

		Sub-elite	385.8 ± 70.1†	383.4 ± 70.0	0.003 (0.033)	

	U15	Elite	427.1 ± 14.01†	423.7 ± 14.3	< 0.0001 (0.241)	
		Sub-elite	417.2 ± 25.2†	414.4 ± 25.9	0.001 (0.108)	

	U17	Elite	423.1 ± 12.0†	420.1 ± 12.4	< 0.0001 (0.245)	
		Sub-elite	417.1 ± 17.1†	413.1 ± 16.5	< 0.0001 (0.238)	

Zone 3 (m)	U13	Elite	181.1 ± 10.2†	178.8 ± 10.2	0.002 (0.228)	Format: F_1.66_ = 106.576; p < 0.0001; ES = 1.246 Age: F_2.66_ = 4.965; p = 0.01; ES = 0.339 Level: F_1.66_ = 2.902; p = 0.093; ES = 0.167 Format × Age: F_2.66_ = 4.323; p = 0.017; ES = 0.310 Format × Level: F_1.66_ = 2.987; p = 0.089; ES = 0.171 Age × Level: F_2.66_ = 10.234; p < 0.0001; ES = 0.517 Format × Age × Level: F_2.66_ = 3.498; p = 0.036; ES = 0.269
		Sub-elite	178.3 ± 17.1	176.9 ± 17.3	NS	

	U15	Elite	188.5 ± 16.1†	185.4 ± 14.5	<0.0001 (0.201)	
		Sub-elite	183.2 ± 7.7†	178.8 ± 8.3	<0.0001 (0.543)	

	U17	Elite	162.9 ± 10.3†	160.7 ± 10.9	0.003 (0.212)	
		Sub-elite	187.0 ± 7.3†	181.9 ± 7.2	< 0.0001 (0.703)	

Zone 4 (m)	U13	Elite	5.7 ± 1.1	5.6 ± 0.9	NS	Format: F_1.66_ = 14.117; p < 0.0001; ES = 0.439 Age: F_2.66_ = 0.857; p = 0.424; ES = 0 Level: F_1.66_ = 4.425; p = 0.039; ES = 0.224 Format × Age: F_2.66_ = 0.294; p = 0.747; ES = 0 Format × Level: F_1.66_ = 1.107; p = 0.297; ES = 0.039 Age × Level: F_2.66_ = 0.256; p = 0.775; ES = 0
		Sub-elite	4.8 ± 0.9	4.8 ± 1.0	NS	

	U15	Elite	5.9 ± 1.2†	5.3 ± 0.9	0.012 (0.563)	
		Sub-elite	5.3 ± 1.1	5.1 ± 0.8	NS	

	U17	Elite	5.8 ± 1.0	5.4 ± 1.0	NS	
		Sub-elite	5.5 ± 1.2	5.1 ± 1.1	NS	

Note: Values are given as means ± SD; stationary/walking (zone 1): 0 to < 7.0 km.h^−1^, low-intensity running (zone 2): 7.0 to < 14.0 km. h^−1^, medium-intensity running (zone 3): 14.0 to < 18.0 km.h^−1^, and high-intensity running (zone 4); for other abbreviations see Table 1; NS: not significant

† : Significantly different from 4-a-side SSGs.

There was a significant interaction between format × level for HRpeak (%HR_max_) and HRmean (%HR_max_) (Table 4), 4-a-side showed higher values than 3-a-side in either U13 (all p < 0.05 for both competitive levels), U15 (all p < 0.05 for both competitive levels), or U17 (elite: all p < 0.0001; sub-elite (only HRpeak): p = 0.030) (Table 4).

**TABLE 4:** Physiological responses during different soccer small-sided game formats in elite and sub-elite players (n = 72).

Variables	Age	Level	Format		Within sub-group comparison p (ES)	ANOVA

			3-a-side	4-a-side		
HR_peak_	U13	Elite	186.8 ± 4.1	190.9 ± 3.0	–	–
		Sub-elite	189.5 ± 2.5	191.5 ± 2.5	–	

	U15	Elite	186.3 ± 2.8	190.6 ± 2.5	–	
		Sub-elite	188.3 ± 3.5	190.2 ± 2.7	–	

	U17	Elite	186.3 ± 2.9	190.3 ± 2.7	–	
		Sub-elite	189.2 ± 3.3	190.3 ± 2.3	–	

HR_peak_(%HR_max_Theo)	U13	Elite	93.6 ± 2.1	95.7 ± 1.5‡	< 0.0001 (1.151)	Format: F_1.66_ = 181.301; p < 0.0001; ES = 0.733 Age: F_2.66_ = 3.182; p = 0.048; ES = 0.088 Level: F_1.66_ = 3.466; p = 0.068; ES = 0.050 Format × Age: F_2.66_ = 0.530; p = 0.591; ES = 0 Format × Level: F_1.66_ = 33.392; p < 0.0001; ES = 0.336 Age × Level: F_2.66_ = 0.278; p = 0.758; ES = 0 Format × Age × Level: F_2.66_ = 0.219; p = 0.804; ES = 0
		Sub-elite	95.0 ± 1.3	96.0 ± 1.2‡	< 0.0001 (0.799)	

	U15	Elite	94.1 ± 1.5	96.3 ± 1.4‡	< 0.0001 (1.516)	
		Sub-elite	95.0 ± 1.8	95.9 ± 1.4‡	< 0.0001 (0.558)	

	U17	Elite	94.7 ± 1.5	96.7 ± 1.4‡	< 0.0001 (1.378)	
		Sub-elite	96.2 ± 1.7	96.8 ± 1.2‡	0.030 (0.408)	

HR_mean_	U13	Elite	156.5 ± 5.4	162.5 ± 3.6	–
		Sub-elite	156.3 ± 6.5	159.8 ± 4.4	–	–

	U15	Elite	156.6 ± 4.6	161.6 ± 3.8	–	
		Sub-elite	156.2 ± 5.7	159.6 ± 4.8	–	

	U17	Elite	156.0 ± 4.3	161.8 ± 3.2	–	
		Sub-elite	160.9 ± 4.0	160.6 ± .5.5	–	

HR_mean_ (%HR_max_Theo)	U13	Elite	78.5 ± 2.7	81.5 ± 1.8‡	< 0.0001 (1.307)	Format: F_1.66_ = 46.305; p < 0.0001; ES = 0.412 Age: F_2.66_ = 4.468; p = 0.015; ES = 0.119 Level: F_1.66_ = 0.116; p = 0.734; ES = 0 Format × Age: F_2.66_ = 1.014; p = 0.368; ES = 0.030 Format × Level: F_1.66_ = 9.165; p = 0.004; ES = 0.122 Age × Level: F_2.66_ = 1.416; p = 0.250; ES = 0.041 Format × Age × Level: F_2.66_ = 1.514; p = 0.228; ES = 0.044
		Sub-elite	78.4 ± 3.2	80.1 ± 2.3‡	0.018 (0.61)	

	U15	Elite	79.1 ± 2.4	81.7 ± 2.0‡	0.001 (1.177)	
		Sub-elite	78.8 ± 2.9	80.5 ± 2.4‡	0.017 (0.639)	

	U17	Elite	79.3 ± 2.2	82.3 ± 1.6‡	< 0.0001 (1.56)	
		Sub-elite	81.8 ± 2.1	81.6 ± 1.8	NS	

BLa (mmol.L^-1^)	U13	Elite	4.6 ± 0.2†	4.5 ± 0.2	0.001 (0.258)	Format: F_1.66_ = 54.624; p < 0.0001; ES = 0.888 Age: F_2.66_ = 0.607; p = 0.548; ES = 0 Level: F_1.66_ = 7.466; p = 0.008; ES = 0.308 Format × Age: F_2.66_ = 0.175; p = 0.840; ES = 0 Format × Level: F_1.66_ = 0.204; p = 0.026; ES = 0.249 Age × Level: F_2.66_ = 0.011; p = 0.989; ES = 0 Format × Age × Level: F_2.66_ = 0.952; p = 0.391; ES = 0
		Sub-elite	4.6 ± 0.1†	4.6 ± 0.1	0.004 (0.315)	

	U15	Elite	4.5 ± 0.1†	4.5 ± 0.1	0.001 (0.444)	
		Sub-elite	4.6 ± 0.1	4.6 ± 0.1	NS	

	U17	Elite	4.5 ± 0.1†	4.5 ± 0.1	< 0.0001 (0.905)	
		Sub-elite	4.6 ± 0.1	4.6 ± 0.1	NS	

Note: Values are given as means ± SD; HR_peak_: heart rate peak; %HR_max_Theo: percentage of the theoretical maximum heart; HR_mean_: heart rate mean; BLa: blood lactate; For other abbreviations see Table 1; ES: effect size; NS: not significant;

† : Significantly different from 4-a-side SSGs.

‡ : Significantly different from 3-a-side SSGs.

For BLa, the 3-a-side showed higher concentration than 4-a-side in elites across all age categories (all p < 0.05) and U13-sub-elite (p = 0.004) (Table 4). There was a significant main effect for levels with sub-elite having higher BLa concentration than elite in U17 in the 4-a-side (p = 0.044; ES = 1.273) (Table 4).

## DISCUSSION

We aimed to compare the time-motion physical performance and physiological responses of young soccer players of different age categories and competitive levels during fixed pitch size SSGs performed under different formats.

To our knowledge, this is the first study investigating the effect of the competitive level on physical performance and physiological responses in different SSG formats (i.e., different-size players but with fixed pitch size). The finding that elite players covered larger TD and distances in the first and fourth (high-intensity running) speed zones aligns with previous research emphasizing the superior physical attributes of elite players [36]. Notably, previous studies have identified better physical performances (i.e., jumping, agility) in elite than sub-elite players of the same age [22]. Interestingly, BLa values were higher in U17-sub-elite players compared to their elite counterparts in the 4-a-side SSGs. This may indicate variations in the use of the anaerobic energy system and lactate clearance. Variations in fitness levels, training status, and the capacity to clear and tolerate lactate during high-intensity activities may be to blame for this [37]. According to earlier research, elite players frequently exhibit more effective lactate clearance mechanisms, which improves performance in repeated high-intensity efforts [37]. According to Reilly et al. [38], the U17 age group marks a critical time in a player’s development when the physiological and physical distinctions between elite and sub-elite players may intensify. These results confirm the significance of taking into account physiological reactions in addition to physical performance measurements in order to fully comprehend the variations between elite and sub-elite soccer players during SSGs. Moreover, these variations highlight the importance of targeted training and conditioning programs in closing the performance gap between sub-elite and elite levels, particularly in the critical developmental stage of U17 players.

Changing the game format significantly affected the players’ physical performances and physiological responses during the SSGs performed on fixed pitch size. As mentioned above, the 3-a-side induced larger TD and distances covered at different speeds, and better Vpeak than the 4-a-side. These findings contrast previous research reporting no differences between both game formats in TD and different speed zones recorded in recreational youth soccer [16]. In our study, the pitch size was constant in both game formats, which may impose a longer overall TD in smaller team sizes (i.e., 3-a-side). In this regard, it has been highlighted that the dimensions of the playing area per player might play a significant role in accounting for the observed discrepancies in both physical and physiological performances [39]. This suggests that adjusting the pitch size in SSGs may impact the players’ performance metrics (e.g., TD, distances covered at different speeds, Vpeak). Our findings suggest that the metrics’ variables are sensitive to changes in number of players during fixed pitch size SSGs. However, further investigation into the physiological, acceleration/deceleration, tactical, and technical aspects of both formats could provide a more comprehensive understanding of the observed differences in player performance to control the intensity of SSGs rather than isolating one variable.

Interestingly, our results extend previous study reporting higher BLa in the 3-a-side SSGs compared to the 4-a-side SSGs [16]. It has been reported a decline in BLa with an increasing number of players, while maintaining a constant pitch size SSGs [40]. Thus, it is conceivable to suggest that increasing the number of players, may contribute significantly to the difference of BLa between the two formats. The differences in Bla are in agreement with the greater distance covered at medium to high speeds, indicating a high anaerobic energy turnover, leading to the accumulation of lactate in the blood [31]. The multifaceted nature of peak lactate level is crucial, as the total high-speed distances does not solely determine it. Factors such as soccer-specific motions and ball contacts also significantly contribute to its variation [31].

Our data also showed a game format effect on HRpeak(%HR_max_) and HRmean(%HR_max_), with higher values observed during the 4-aside SSGs than the 3-a-side SSGs. These results do not align with previous reports [16], where no significant differences were noted between the two format games for HR variables. The discrepancy in our findings may be attributed to the increased tactical complexity associated with larger team sizes. Larger teams often introduce greater tactical intricacies, requiring players to adapt to dynamic game situations continuously [41]. The higher engagement of different energy systems likely contributes to the observed differences in HR responses between 3- and 4-a-side SSGs in our study. Given how various energy systems may be used in each format, the higher HR values in 4-a-side SSGs may indicate a higher aerobic component, while the increased physical demands (i.e., medium to high-intensity running) and BLa levels in 3-a-side SSGs may point to a greater dependence on anaerobic energy systems [16].

With regard to the influence of age categories on physical performances, previous studies have indicated that players’ physical response during different SSGs formats and official matches is contingent upon age category [13, 25]. These findings align with our study’s results, demonstrating a higher TD covered by sub-elite older players (i.e., U15 and U17) compared to their younger counterparts (i.e., U13) in both SSG formats. The existing body of research on youth soccer players’ performance in SSGs reveals nuanced age-related differences. Silva et al. [42] emphasized the tendency for older players to exhibit more accurate decision-making during games. Clemente et al.’s [43] systematic review highlighted significant disparities in the tactical behavior of players from different age groups in SSGs, with younger players often prioritizing ball chasing and individual actions over maintaining balanced space coverage [11]. Folgado et al. [44] noted that younger players tended to explore the length more than the width of the pitch, while Clemente et al. [45] demonstrated age-related variations in the area occupied by players during a four vs. four plus goalkeeper SSGs, with U18 players occupying the largest area, followed by U15 and U13 players. The adaptation of performance behaviors, dispersion while attacking, and the influence of tactical knowledge were also found to be age-dependent aspects [46, 47]. Likewise, the aforementioned studies emphasized that as players become more experienced and competent, they adjust their performance behaviors to the space covered and game dynamics, exploring available playing space. Furthermore, although not directly assessed, the study suggested that high level of maturity might play a role in the observed differences, with older players (i.e., U15 and U17) potentially benefiting from increased anthropometric characteristics (e.g. height, leg length, step length) and physical capabilities (e.g., TD, speed, strength) than U13 ones [48]. Future research is recommended to include direct measures of maturity to substantiate these observations. Surprisingly, our findings showed that U13 and U15 elite groups covered more distances at zone-3 than their older counterparts, despite the fact that all age categories presented similar TD, Vpeak and distances covered at different speeds (i.e., zone-1 and zone-4). This result may be related to the older athletes’ sparse tactical knowledge of fully exploring pitch size and their keener ability of scanning/reading the game, exhibiting higher cooperation among team players as a result of being more experienced [12]. For this reason, whenever possible, coaches should consider the area per player adjustments to better align training regimens with the dynamic demands of competitive game [49].

“We advise practitioners to ensure that training tasks within SSGs are tailored to different age categories, as players of varying ages experience distinct external and internal loads. Using similar tasks across age groups can hinder the effectiveness of the training and the overall objectives of the session, ultimately impacting the longterm development of players. Practitioners should consider the different training stages and adapt the intensity and type of stimuli to suit the condition of players at each level, focusing on their medterm to long-term growth.

This study acknowledges certain limitations that warrant consideration by researchers and coaches when interpreting the findings. One notable limitation is the exclusion of acceleration and deceleration data from the analysis. The decision to omit this data was made to streamline the focus on some specific physical responses during SSGs. However, it is crucial to recognize that acceleration and deceleration play significant roles in soccer performance and may contribute valuable insights. Future research endeavors could explore the impact of acceleration and deceleration patterns on the overall dynamics of SSGs, providing a more comprehensive understanding of the players’ movement dynamics during these training scenarios. Second, the inability to achieve perfect counterbalancing in the experimental design, which was mitigated by employing a random draw. Additionally, the measurement of maturation was not included, which could provide further insights into the developmental differences among age groups. Measuring maturation provides a more accurate assessment of an athlete’s development stage, allowing for more tailored training interventions that align with their individual growth patterns [50]. For example, the use of bio-banding method, can create more equitable competition and training environments, ensuring that late maturers are not disadvantaged [50, 51].

## CONCLUSIONS

In conclusion, our study provides detailed insights into the physical and physiological responses of young soccer players across different age categories (U13, U15, U17) and competitive levels (elite, subelite) during fixed pitch size SSGs. We found that the 3-a-side format induces higher physical demands compared to the 4-a-side format, particularly among elite players. Additionally, older players (U15, U17) exhibited superior performance metrics compared to younger players (U13). These findings highlight the importance of tailoring training programs based on age and competitive level to optimize player development and performance. Further research should include measures of biological maturation to deepen our understanding of developmental differences.
